# Predictors for regression and progression of intestinal metaplasia (IM): A large population-based study from low prevalence area of gastric cancer (IM-predictor trial)

**DOI:** 10.1371/journal.pone.0255601

**Published:** 2021-08-11

**Authors:** Natsuda Aumpan, Ratha-korn Vilaichone, Bubpha Pornthisarn, Soonthorn Chonprasertsuk, Sith Siramolpiwat, Patommatat Bhanthumkomol, Pongjarat Nunanan, Navapan Issariyakulkarn, Sarita Ratana-Amornpin, Muhammad Miftahussurur, Varocha Mahachai, Yoshio Yamaoka

**Affiliations:** 1 Center of Excellence in Digestive Diseases and Gastroenterology, Faculty of Medicine, Thammasat University, Pathumthani, Thailand; 2 Chulabhorn International College of Medicine (CICM), Thammasat University, Pathumthani, Thailand; 3 Division of Gastroentero-Hepatology, Department of Internal Medicine, Faculty of Medicine, Universitas Airlangga, Surabaya, Indonesia; 4 Department of Environmental and Preventive Medicine, Faculty of Medicine, Oita University, Yufu, Japan; Holbaek Sygehus, DENMARK

## Abstract

**Background:**

Gastric intestinal metaplasia (IM) can lead to gastric cancer. Until now, there have been limited studies of predictors for regression and progression of IM. This study aimed to determine risk factors associated with regression or progression of IM for guiding proper management and prevention of gastric cancer.

**Methods:**

2,025 patients undergoing gastroscopy in Thammasat University Hospital, Thailand were enrolled during September 2017-August 2019. Patients’ data including baseline characteristics, laboratory results, and histopathology of gastric biopsies from University medical database were extensively reviewed.

**Results:**

2,025 patients had mean age of 61.3 years and 44.2% were males. Overall *H*. *pylori* prevalence was 47.5%. There were 1,551(76.6%) patients with chronic gastritis and 361(17.8%) with IM. Of 400 patients with chronic gastritis having follow-up endoscopy and repeated gastric biopsies, 104(26%) had persistent *H*. *pylori* infection and 27(26%) developed IM during mean follow-up time of 24 months. Persistent *H*. *pylori* infection was significantly associated with development of IM (OR 3.16, 95%CI 1.56–6.39, p = 0.001). Regression, persistence, and progression of IM were demonstrated in 57.3%, 39.2%, and 3.5% of patients, respectively. Age >65 years, persistent *H*. *pylori* infection, and diabetes mellitus were significantly associated with persistent IM or progression to dysplasia with OR 2.47(95%CI 1.33–4.61, p = 0.004), OR 2.64(95%CI 1.13–6.18, p = 0.025), and OR 2.54(95%CI 1.16–5.54, p = 0.019), respectively. Patients without *H*. *pylori* infection had more IM regression than patients with persistent infection (60.4%vs.39.4%, p = 0.035). Patients with persistent *H*. *pylori* infection significantly had higher IM progression to dysplasia (15.2%vs.2.1%; OR 11.15, 95%CI 1.18–105.24, p = 0.035) than noninfected. During 24 months of study, 30 patients (1.5%) were diagnosed with gastric cancer.

**Conclusion:**

Regression of IM could be achieved by successful *H*. *pylori* eradication. Persistent *H*. *pylori* infection was significantly associated with development and progression of IM to dysplasia. Age >65 years and diabetes mellitus were also significant predictors for IM progression.

## Introduction

Gastric cancer ranks fourth highest for global cancer-related mortality. The recent study has reported more than 700,000 deaths and 1 million new cases in 2020 [1]. The burden of gastric cancer was disproportionately higher in Eastern Asia and Eastern Europe, whereas Northern Europe and Northern America had lower incidence rates [2]. Frequently diagnosed at an advanced stage, gastric cancer is considered as one of the most aggressive cancers contributing to ominous prognosis. According to Lauren classification, gastric adenocarcinomas, the most common type of gastric cancer, were categorized into two histologic subtypes, intestinal and diffuse type [3]. The diffuse-type gastric cancer arises from invading tumour cells through defects of intercellular adhesion molecules, while the intestinal type occurs through sequential development of precancerous lesions in the multistage carcinogenesis [4]. The precancerous cascade is primarily driven by *Helicobacter pylori (H*. *pylori)* infection which can turn persistent gastric mucosal inflammation into atrophic gastritis, intestinal metaplasia, dysplasia, and eventually adenocarcinoma [5].

Gastric intestinal metaplasia (IM) is a premalignant lesion of intestinal-type gastric cancer. This metaplastic change develops as a result of chronic inflammation of gastric epithelial cells induced by bacterial factors combined with host immune response. Sustained inflammatory insult induces loss of gastric glands, and subsequent replacement of gastric mucosa by intestinal epithelium [5]. The annual incidence of gastric cancer was 0.25% for patients with IM and 1.8% in 10 years of follow-up after diagnosis of IM [6]. Previous studies revealed conflicting evidence whether IM could regress or progress over a period of time. Some studies demonstrated reversal of IM after *H*. *pylori* eradication [7, 8], while others reported persistence of IM after treatment [9, 10]. Moreover, the prior study indicated factors (e.g., persistent *H*. *pylori* infection, age >45 years, duodenal ulcers) significantly associated with progression of IM [11]. In the country with low prevalence of gastric cancer, the latest guideline stated that there were still research gaps about management of gastric IM after diagnosis [12].

Until now, there have been limited studies examining the impact of surveillance program on outcomes of IM after diagnosis. The guideline recommended using risk stratification to identify patients with higher risk for gastric cancer and suggested that endoscopic surveillance should be performed in these high-risk groups [12]. In Thailand, a country with low prevalence of gastric cancer, there were limited data about associated risk factors on progression of IM. This study aimed to determine risk factors related to regression or progression of IM after diagnosis.

## Materials and methods

### Study design

This retrospective cohort study was conducted at Thammasat University Hospital between September 2017 and August 2019. The inclusion criteria were Thai patients aged more than 15 years old who had indication for endoscopic evaluation such as chronic dyspepsia, chronic abdominal pain, iron deficiency anaemia [13]. All patients underwent upper gastrointestinal endoscopy and gastric biopsies were performed during the endoscopic procedure. Patients with pathology reports of chronic gastritis, atrophic gastritis, intestinal metaplasia, dysplasia, or adenocarcinoma were included in this study, while others without gastric biopsies were excluded. Demographic data, comorbidity, clinical presentation, laboratory results including a complete blood count and a comprehensive metabolic panel, endoscopic and pathology results, current *H*. *pylori* infection status, duration of *H*. *pylori* infection were extracted from medical database and extensively reviewed.

The primary aim of this study was to determine risk factors associated with regression or progression of IM for guiding proper management and prevention of gastric cancer.

### The endoscopic gastric mapping

Each patient in this study underwent upper gastrointestinal endoscopy, which was performed by sufficient air insufflation, gastric mucosal cleaning, and minimum examination time at least 7 minutes to ensure adequate mucosal visualisation [4]. The gastric areas composed of greater curvature, lesser curvature, antrum, pylorus, incisura, fundus, and cardia were thoroughly inspected and photographically documented. In this study, gastric mapping required a minimum of 3 biopsies from antrum, corpus, and incisura. If there was a visible lesion compatible with precancerous lesion from conventional or image-enhanced endoscopy, 2 additional biopsies would be performed at lesion. Rapid urease test was performed using the first antral biopsy, and histopathology using two biopsies from antrum and body.

### Histopathology of *H*. *pylori* infection

*H*. *pylori* infection can be directly diagnosed by histopathological examination. Hematoxylin and eosin (H&E) is a standard staining commonly used to identify *H*. *pylori* as gram-negative, spiral-shaped bacteria in the gastric mucus layer, while Giemsa stain is a special staining method which help identify blue-stained *H*. *pylori* more easily.

### Definition

***H*. *pylori* infection** was defined as either positive rapid urease test or detection of curved rod-shaped bacteria by histopathological examination of patients’ most recent gastric biopsy.

**Persistent *H*. *pylori* infection** was defined as persistent detection (>24 months) of *H*. *pylori* infection by either positive rapid urease test or histopathological result from first diagnosis to the most recent gastric biopsy performed.

**Chronic gastritis** was defined as lamina propria containing more than 2 to 5 of mononuclear leukocytes, e.g., lymphocytes, plasma cells, and macrophages per high power microscopic field (x40 objective) [14]. In this study, chronic gastritis included only non-atrophic gastritis.

**Atrophic gastritis** was defined as a loss of gastric glands resulted from chronic gastric mucosal inflammation [5].

**Intestinal metaplasia (IM)** was defined as the replacement of original gastric glands and foveolar epithelium by intestinal epithelial cells. IM was classified by histopathology into 2 types, complete and incomplete IM [15]. Complete IM is composed of small intestinal epithelial linings with well-defined brush border and mature goblet cells, while incomplete IM comprises slightly architectural-distorted crypts with multiple intracytoplasmic mucin droplets of variable size and absence of a brush border resembling a colonic epithelium [15].

**Dysplasia** was defined as disorganized architecture of gastric glandular structures composed of hyperchromatic, elongated nuclei without extension beyond basement membrane [16].

**Progression of IM** was defined as histologic progression from IM to dysplasia on repeated gastric biopsies over time [17].

**Persistence of IM** was defined as histologic persistence of IM on repeated gastric biopsies over time.

**Regression of IM** was defined as histologic regression from IM to normal gastric mucosa, non-atrophic gastritis, or atrophic gastritis on repeated gastric biopsies over time [17].

### Statistical analysis

All data were analysed by using SPSS version 22 (SPSS Inc., Chicago, IL, USA) and Stata version 16.0 (StataCorp, College Station, Texas 77845, USA). Categorical variables were analysed by Fisher’s exact test, or Chi-square test where appropriate. Continuous variables were analysed by using Student’s t-test and reported as mean ± standard deviation (SD). Univariate analysis was performed to identify significant demographic or other risk factors affecting progression from chronic gastritis to IM, and from IM to dysplasia. Every variable with a p value of less than 0.05 by univariate analysis were entered into the multivariate analysis. Multivariate logistic regression analysis was then performed, with backward elimination method, to identify independent predictors of the end point: persistence or progression of IM. All tests were two-sided and p values of less than 0.05 were considered statistically significant. Cox proportional hazards model was performed to test for factors influencing IM persistence or progression at a particular point in time. Kaplan-Meier curve was plotted for probability of persistence of progression of IM between persistent vs. no *H*. *pylori* infection, and diabetic vs. non-diabetic patients.

The study obtained ethical approval by the Human Research Ethics Committee of Thammasat University, Thailand and was conducted according to the good clinical practice guideline, as well as the Declaration of Helsinki. All data had been fully anonymized before they were accessed. Waiver of documentation of informed consent was issued by the Ethics Committee owing to no greater than minimal risk to study subjects. The project number of ethical approval was MTU-EC-IM-6-160/62.

## Results

**Fig 1:**
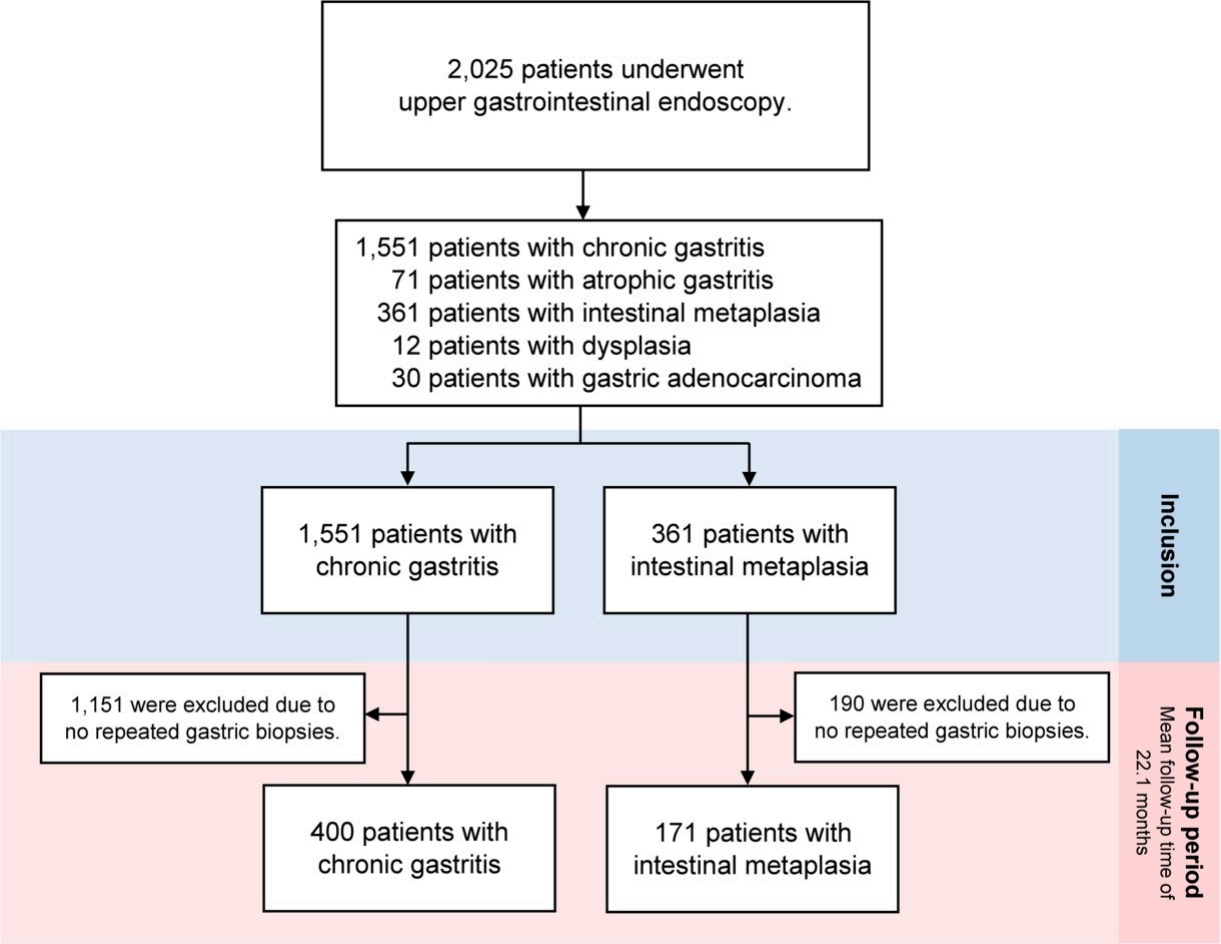
The study flow chart.

A total of 2,025 Thai patients [895 men and 1,130 women; mean age 61.3 ± 13.4 (range 16–96) years] were included in this study. All patients had indications for upper gastrointestinal endoscopy as stated in the national dyspepsia guideline by The Gastroenterological Association of Thailand (GAT) [13]. The most common indications were dyspepsia (58.7%). Classified by histopathology results, the overall prevalence of intestinal metaplasia was 17.8%. The overall prevalence of *H*. *pylori* infection in this study was 47.5%. The study flow chart was demonstrated in Fig 1.

### Demographic data and risk factors for the development of intestinal metaplasia

Demographic data and associated risk factors of 1,551 patients with chronic gastritis and 361 patients with intestinal metaplasia were included for univariate and multivariate analyses (Tables 1 and 2). There were 42% of patients with chronic gastritis and 66.5% of patients with IM having *H*. *pylori* infection. Age >50 years, current *H*. *pylori* infection, and hypertension as underlying disease were significantly associated with IM with OR 1.67 (95% CI 1.15–2.42, p = 0.007), OR 2.70 (95% CI 2.11–3.47, p <0.001), and OR 1.31 (95% CI 1.02–1.69, p = 0.036), respectively. Males, pulmonary disease as comorbidity, smoking, and alcohol use were risk factors associated with IM in the univariate analysis. However, these factors could not reach statistical significance in the multivariate analysis. Body mass index (BMI) and family history of gastric cancer were not different between groups.

**Table 1:** Univariate analysis of associated risk factors and odds ratio in patients with chronic gastritis and intestinal metaplasia.

Risk factors	Chronic gastritis (N = 1,551)	Intestinal metaplasia (N = 361)	Odds ratio (95% CI)	P-value
**Male (%)**	**649 (41.8%)**	**190 (52.6%)**	**1.54(1.23–1.94)**	**<0.001**
**Age (years ± SD)**	**60.4 ± 13.4**	**65.4 ± 12.8**	**N/A**	**<0.001**
**>50 years**	**1,231 (79.4%)**	**320 (88.6%)**	**2.03(1.43–2.87)**	**<0.001**
BMI (kg/m^2^ ± SD)	23.9 ± 4.3	23.9 ± 4.7	N/A	0.983
***H*. *pylori* infection (%)**	**652 (42.0%)**	**240 (66.5%)**	**2.74(2.15–3.48)**	**<0.001**
Underlying diseases				
None	364 (23.5%)	80 (22.2%)	1.08 (0.82–1.42)	0.596
Diabetes mellitus	317 (20.4%)	77 (21.3%)	1.06 (0.80–1.40)	0.706
**Hypertension**	**619 (39.9%)**	**172 (47.6%)**	**1.37 (1.09–1.73)**	**0.007**
Dyslipidemia	558 (36.0%)	131 (36.3%)	1.01 (0.80–1.29)	0.912
Chronic kidney disease	100 (6.4%)	28 (7.8%)	1.22 (0.79–1.89)	0.371
Cardiovascular disease	131 (8.4%)	42 (11.6%)	1.43 (0.99–2.06)	0.058
Cirrhosis and hepatitis	268 (17.3%)	67 (18.6%)	1.09 (0.81–1.47)	0.564
**Pulmonary diseases**	**45 (2.9%)**	**18 (5.0%)**	**1.76 (1.00–3.07)**	**0.048**
Neurological disorders	94 (6.1%)	31 (8.6%)	1.46 (0.95–2.22)	0.082
Rheumatic diseases	90 (5.8%)	20 (5.5%)	0.95 (0.58–1.57)	0.847
Malignancy	124 (8.0%)	37 (10.2%)	1.31 (0.89–1.93)	0.166
Family history of gastric cancer (%)	9 (0.6%)	5 (1.4%)	2.41 (0.80–7.22)	0.117
**Smoking (%)**	**136 (9.3%)**	**54 (15.5%)**	**1.78 (1.27–2.50)**	**0.001**
**Alcohol (%)**	**211 (14.5%)**	**82 (23.5%)**	**1.81 (1.36–2.41)**	**<0.001**

Univariate analysis was performed to determine odds ratio, 95% confidence interval, and p-value.

Student’s t-test was performed to determine difference of the mean of age and body mass index.

**Table 2:** Multivariate analysis of associated risk factors and odds ratio in patients with chronic gastritis and intestinal metaplasia.

Risk factors	Odds ratio (95% CI)	p-value
Male	1.26 (0.95–1.66)	0.108
**Age >50 years**	**1.67 (1.15–2.42)**	**0.007**
***H*. *pylori* infection**	**2.70 (2.11–3.47)**	**<0.001**
**Hypertension**	**1.31 (1.02–1.69)**	**0.036**
Pulmonary diseases	1.56 (0.86–2.83)	0.144
Smoking	1.18 (0.77–1.81)	0.441
Alcohol use	1.43 (0.98–2.08)	0.061

Multivariate analysis was performed to determine odds ratio, 95% confidence interval, and p-value.

### Follow-up of chronic gastritis

**Fig 2:**
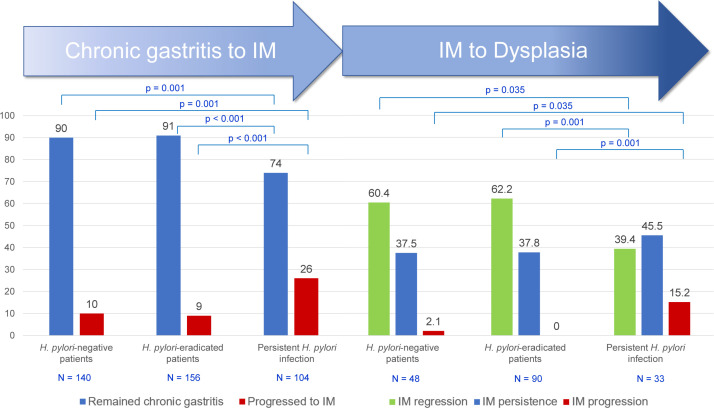
Progression of chronic gastritis and IM according to persistence of *H*. *pylori* infection.

Of 400 patients with chronic gastritis having a follow-up endoscopy and repeated gastric biopsies, 260 had *H*. *pylori* infection and 232 (89.2%) were treated by antibiotics. The most commonly used regimens for *H*. *pylori* treatment were triple therapy (57.3%), concomitant therapy (18.5%), and bismuth quadruple regimen (14.7%). The eradication rate was 60%. During the mean follow-up time of 24 months, there were 140 (35%) patients without prior *H*. *pylori* infection, 156 (39%) with eradicated-*H*. *pylori* infection, and 104 (26%) with persistent *H*. *pylori* infection. Persistent *H*. *pylori* infection was significantly associated with progression from chronic gastritis to IM compared to patients without prior infection (26% vs. 10%; OR 3.16, 95% CI 1.56–6.39, p = 0.001) (Fig 2). The progression rates from chronic gastritis to IM were not different between *H*. *pylori*-eradicated and *H*. *pylori*-negative patients (9% vs. 10%; OR 0.89, 95% CI 0.41–1.93, p = 0.763). There was no difference of a complete blood count, and a comprehensive metabolic panel between group of chronic gastritis without progression and a group with progression to IM (S1 Table).

### Follow-up of gastric intestinal metaplasia

Of 361 patients with IM, 171 had a follow-up endoscopy with repeated gastric biopsies. Regression, persistence, and progression of IM were demonstrated in 98 (57.3%), 67 (39.2%), and 6 (3.5%) patients, respectively. The prevalence of *H*. *pylori* infection at baseline of both IM regression group (70.4%) and IM non-regression group (74%) was not different. Of 123 *H*. *pylori*-infected patients with IM, 95.1% received antibiotic treatment. The most commonly used regimens were triple therapy (53%), concomitant therapy (21.4%), and bismuth quadruple regimen (18.8%). The eradication rate was 73.2%. There were 48 (28.1%) patients without prior *H*. *pylori* infection, 90 (52.6%) with eradicated-*H*. *pylori* infection, and 33 (19.3%) with persistent *H*. *pylori* infection. IM non-regression group had persistent *H*. *pylori* infection (27.4%) more than the regression group (13.3%). The mean follow-up time in the group of IM regression (21.9 months) was almost the same as the group of IM persistence or progression (22.2 months).

### Risk factors for progression of intestinal metaplasia

**Fig 3:**
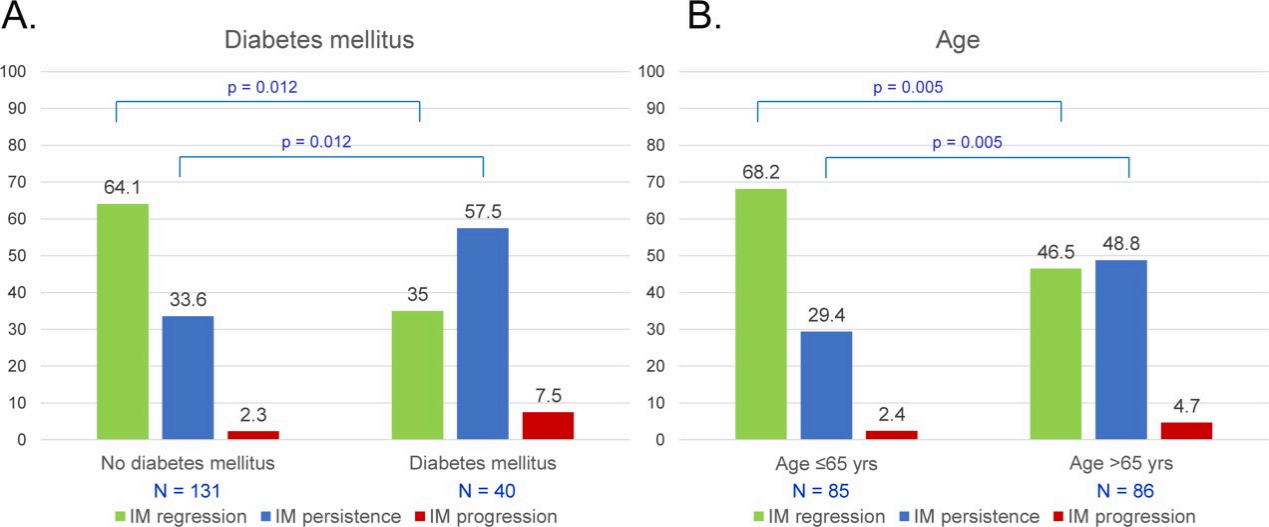
Progression of IM according to diabetes mellitus (A) and age (B).

Age >65 years was significantly related to IM persistence or progression with OR 2.47 (95% CI 1.33–4.61, p = 0.004) (Table 3). In multivariate analysis, persistent *H*. *pylori* infection, and diabetes mellitus were significantly associated with persistent IM or progression to dysplasia with OR 2.64 (95% CI 1.13–6.18, p = 0.025), and OR 2.54 (95% CI 1.16–5.54, p = 0.019), respectively (Table 4). Patients without *H*. *pylori* infection significantly had more IM regression than patients with persistent infection (60.4% vs. 39.4%, p = 0.035). Persistently *H*. *pylori*-infected patients significantly had more IM progression to dysplasia (15.2% vs. 2.1%; OR 11.15, 95% CI 1.18–105.24, p = 0.035) than patients without previous infection (Fig 2, S2 Table). Patients with successful *H*. *pylori* eradication demonstrated comparable IM regression (62.2% vs. 60.4%, p = 0.35) and progression (0% vs. 2.1%, p = 0.35) as patients without prior infection. Patients with diabetes mellitus significantly had more persistent IM than patients without this condition (57.5% vs. 33.6%; OR 2.71, 95% CI 1.24–5.90, p = 0.012) (Fig 3A, S2 Table). Patients aged >65 years significantly had more persistent IM than the younger (48.8% vs. 29.4%; OR 2.49, 95% CI 1.31–4.73, p = 0.005) (Fig 3B, S2 Table). Gender, BMI, type of IM, family history of gastric cancer, alcohol use, and smoking were not significantly different between groups (Table 3). There was no difference of complete blood count, creatinine, and lipid profile between group of IM regression and non-regression. However, plasma glucose (128 ± 39 vs. 106 ± 20 mg/dl, p = 0.008) and hemoglobin A1C levels (6.8 ± 1.3 vs. 6.0 ± 0.7%, p = 0.012) of IM non-regression group were significantly higher than the regression group (S3 Table).

**Table 3:** Demographic data of patients with IM classified by progression of IM.

Risk factors	IM regression (N = 98)	IM non-regression* (N = 73)	Odds ratio (95% CI)	P-value
Gender (%Male)	58 (59.2%)	39 (53.4%)	0.79 (0.43–1.46)	0.452
**Age (years ± SD)**	**63.0 ± 11.3**	**67.2 ± 12.5**	**N/A**	**0.023**
**>65 years**	**40 (40.8%)**	**46 (63.0%)**	**2.47 (1.33–4.61)**	**0.004**
BMI ± SD (kg/m^2^)	24.4 ± 5.2	24.8 ± 6.0	N/A	0.720
*H*. *pylori* infection	69 (70.4%)	54 (74.0%)	1.20 (0.61–2.36)	0.608
**Persistent infection (%)**	**13 (13.3%)**	**20 (27.4%)**	**2.47 (1.13–5.37)**	**0.023**
Mean follow-up time (months)	21.9 ± 18.1	22.2 ± 17.6	N/A	0.922
Type of IM				
Complete IM	85 (86.7%)	63 (86.3%)	1	-
Incomplete IM	13 (13.3%)	10 (13.7%)	1.04 (0.43–2.52)	0.935
Underlying disease				
None	25 (25.5%)	12 (16.4%)	0.57 (0.27–1.24)	0.157
**Diabetes mellitus**	**14 (14.3%)**	**26 (35.6%)**	**3.32 (1.58–6.97)**	**0.002**
Hypertension	37 (37.8%)	38 (52.1%)	1.79 (0.97–3.31)	0.063
**Dyslipidemia**	**28 (28.6%)**	**35 (47.9%)**	**2.30 (1.22–4.34)**	**0.010**
Chronic kidney disease	8 (8.2%)	8 (11.0%)	1.39 (0.49–3.88)	0.536
Cardiovascular disease	7 (7.1%)	5 (6.8%)	0.96 (0.29–3.14)	0.941
Cirrhosis and hepatitis	23 (23.5%)	14 (19.2%)	0.77 (0.37–1.63)	0.501
Pulmonary diseases	7 (7.1%)	3 (4.1%)	0.56 (0.14–2.23)	0.409
Neurological disorders	5 (5.1%)	8 (11.0%)	2.29 (0.72–7.31)	0.162
Rheumatic diseases	6 (6.1%)	3 (4.1%)	0.66 (0.16–2.72)	0.562
Malignancy	8 (8.2%)	11 (15.1%)	2.00 (0.76–5.25)	0.161
Medication use				
Proton pump inhibitor	33 (33.7%)	31 (42.5%)	1.45 (0.78–2.72)	0.241
Aspirin	11 (11.2%)	16 (21.9%)	2.22 (0.96–5.13)	0.062
Statin	31 (31.6%)	28 (38.4%)	1.35 (0.71–2.54)	0.361
Metformin	7 (7.1%)	11 (15.1%)	2.31 (0.85–6.28)	0.102
Family history of gastric cancer (%)	3 (3.1%)	1 (1.4%)	0.44 (0.05–4.32)	0.481
Smoking (%)	18 (18.9%)	8 (11.3%)	0.54 (0.22–1.33)	0.182
Alcohol (%)	27 (28.4%)	14 (19.4%)	0.61 0.29–1.27)	0.184

*IM non-regression = IM persistence or progression to dysplasia.

Univariate analysis was performed to determine odds ratio, 95% confidence interval, and p-value.

Student’s t-test was performed to determine difference of the mean of age, BMI, and follow-up time.

**Table 4:** Multivariate analysis of clinical factors and persistence of IM or progression to dysplasia.

Risk factors	Odds ratio (95% CI)	p-value
**Persistent *H*. *pylori* infection**	**2.64 (1.13–6.18)**	**0.025**
**Diabetes mellitus**	**2.54 (1.16–5.54)**	**0.019**
Dyslipidemia	1.89 (0.96–3.73)	0.067

Multivariate analysis was performed to determine odds ratio, 95% confidence interval, and p-value.

**Fig 4:**
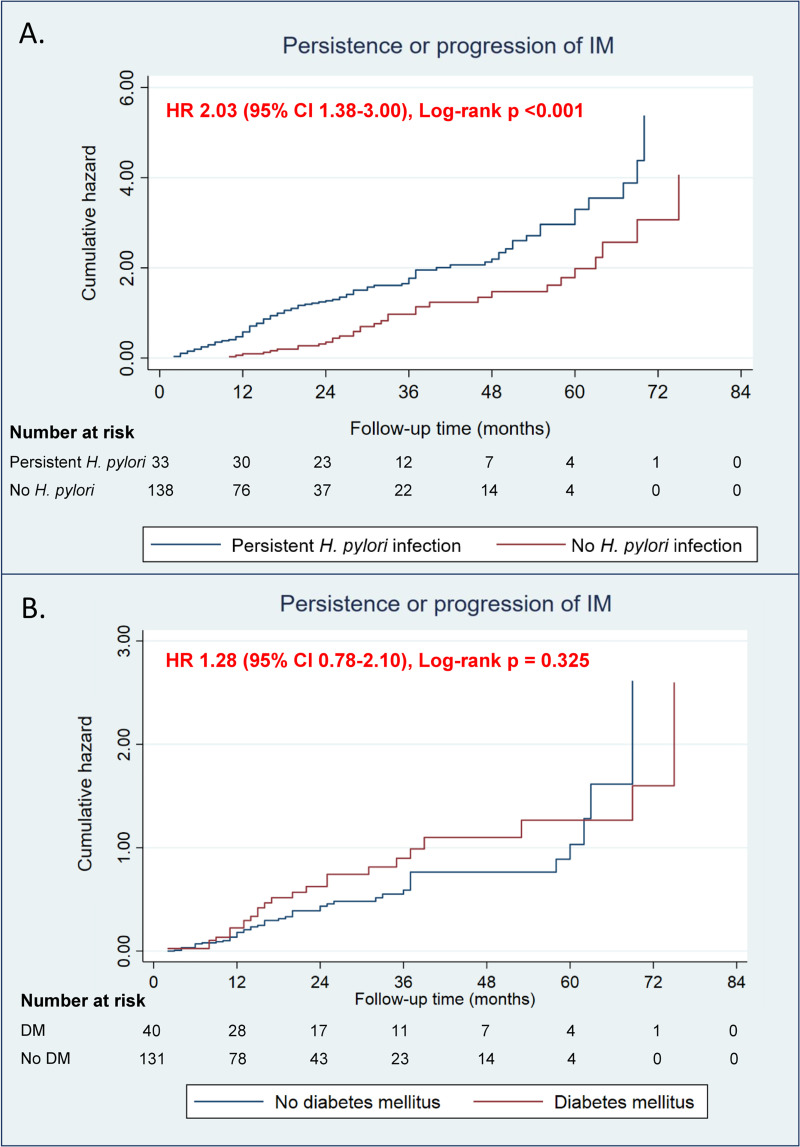
Kaplan-Meier curve for persistence or progression of IM by *H*. *pylori* infection status (A) and diabetes mellitus as comorbidity (B).

Six patients had progression from IM to dysplasia, five of whom had persistent *H*. *pylori* infection for the mean duration of 54 months (range 24–83 months), while one had no prior *H*. *pylori* infection, but a positive family history of gastric cancer. According to the Kaplan-Meier curve, the probability of having IM persistence or progression to dysplasia was approximately two times higher in patients with persistent *H*. *pylori* infection than patients without (hazard ratio [HR] 2.03; 95% CI 1.38–3.00, log-rank p <0.001), while diabetic patients tended to have more IM persistence or progression than non-diabetic patients (HR 1.28; 95% CI 0.78–2.10, log-rank p = 0.325) without statistical significance (Fig 4). During the 24-month study period, there were 30 patients (1.5%) diagnosed with gastric cancer and 11 of them had prior IM along with adenocarcinoma.

## Discussion

Gastric cancer was the leading cause of cancer mortality prior to the 1980s. Since then, the mortality rate from gastric cancer has been steadily decreasing to the fifth rank in 2020 [1]. The decline has been related to successful *H*. *pylori* eradication, improvement in hygiene and sanitation, and less salt use for food preservation [2]. The highest burden of stomach cancer was in East Asia, whereas Western Europe and North America had much lower incidence rates [2]. Thailand, a country in Southeast Asia, has quite the same predicted gastric cancer incidence trend to 2035 as the USA [18]. This study highlighted on risk factors contributing to regression or progression of intestinal metaplasia, an intermediate precancerous gastric lesion. The overall prevalence of IM was 17.8%, which was higher than the prevalence (7.5%) from the large study in the US [19]. This could be explained by higher prevalence of *H*. *pylori* infection in Thailand than the US. Moreover, this study had higher prevalence of *H*. *pylori* infection (47.5%) than the previous report (34.1%) in Thailand [20]. This could be because a large number of patients were referred to our hospital for upper gastrointestinal tract evaluation, and for treatment of *H*. *pylori* eradication failure. Therefore, this could result in higher prevalence of *H*. *pylori* infection than prior reports.

Risk factors associated with the development of IM were identified by previous studies. Both bacterial virulence along with host immune response are factors affecting gastric mucosal inflammation. Age was a demographic variable which had an impact on the development of IM. This study revealed that patients aged over 50 years significantly developed IM on their gastric pathology. This finding is consistent with previous studies indicating increasing prevalence of IM with age, which could be caused by prolonged gastric inflammation [21]. The latest Thai guideline also determined age >50 years as the cut-off point for performing endoscopy in dyspeptic patients because of twofold increase in incidence rate of gastric cancer at this age [13]. Apart from patients’ age, hypertension as comorbidity was independently associated with the development of IM. So far, there has been no previous report about association between gastric premalignant lesion and hypertension. The mechanism of this association still remained unclear. However, the previous study reported the relationship between *H*. *pylori* infection and hypertension in Chinese adults, which presumably resulted from atherosclerosis induced by *H*. *pylori*-related pro-inflammatory cytokines [22]. Lastly, the most important risk factor for the development of IM was current *H*. *pylori* infection since this modifiable factor could be eradicated to cease gastric inflammatory process and subsequent cancer cell differentiation.

The essential question regarding management of gastric precancerous lesion is whether IM can progress or regress during the follow-up period. This study revealed that more than half of patients with IM could have histologic regression to chronic gastritis, while 3.5% of patients had progression to dysplasia. One demographic factor affecting this outcome was an advanced age. People aged >65 years are defined as older adults by the American Geriatrics Society. Older adults were significantly associated with persistent IM or progression to dysplasia. This was possibly due to greater tendency to develop premalignant lesion than the younger. Our finding was different from the result from the prior study in China noting that age >45 years was correlated with IM progression [11]. This might be because of higher prevalence of gastric IM (62.8%) and different definition of IM progression in the mentioned study which included higher IM scores, dysplasia, and cancer as IM progression, while our study included only dysplasia as IM progression. In addition to age, diabetes mellitus as comorbidity was also significantly linked to persistent IM or progression to dysplasia. Various studies described the association between diabetes and gastric cancer via several proposed mechanisms [23, 24]. Hyperglycaemia can generate reactive oxygen species resulting in oxidative DNA damage accumulation, and eventually promote gastric carcinogenesis [25, 26]. Moreover, insulin resistance can induce cell proliferation by overexpression of insulin-like growth factor (IGFs) and heterogeneous expression of IGF-binding proteins (IGFBPs) [27]. Optimal glycaemic control might be beneficial for reducing risk for persistence and progression of IM.

*H*. *pylori* infection plays a pivotal role in the development of gastric cancer. This study demonstrated that persistent *H*. *pylori* infection significantly involved in both steps of gastric carcinogenesis, from chronic gastritis to IM, and from IM to dysplasia. From the first step of precancerous cascade, persistent *H*. *pylori* infection resulted in higher progression rate from chronic gastritis to IM (26%) than the noninfected which was almost equal to the prior study (28%) [28]. Most of studies estimated risk of gastric cancer development from IM [29, 30], whereas only few studies provided the progression rate from IM to dysplasia. One study conducted in high-risk Asian immigrants had very high rate of IM progression to dysplasia (14%) [31], while our study demonstrated a low rate of progression to dysplasia (3.5%) which was comparable to the previous study (2%) in the Netherlands and Norway [32]. However, our IM regression rate was higher (57.3% vs. 32%). This could be due to higher prevalence of *H*. *pylori* infection in Thailand which remarkably decreased from 70.4% to 13.3% by eradication in the regression group, while the other study already had low *H*. *pylori* prevalence at baseline and could not modify this factor as much as ours (infection rate decreased from 26% to 18% after eradication). Moreover, this study reported that *H*. *pylori*-eradicated patients had nearly equal IM regression and progression rates as patients without prior infection. This emphasizes the importance of *H*. *pylori* eradication in order to induce IM regression, and prevent histologic progression.

The strength of our study was that it was a large population-based study demonstrating predictors for IM progression. Moreover, it signified that *H*. *pylori* eradication contributed to IM regression. As this was a retrospective cohort study, it also had some limitations. First, IM was not graded according to the operative link on IM assessment (OLGIM) grading system due to incomplete data about severity grading of IM in some patients. Second, the mean follow-up period was approximately two years, which might be short to evaluate the progression and regression of IM. However, this study demonstrated that two years of persistent *H*. *pylori* infection could result in progression of IM.

In conclusion, persistent *H*. *pylori* infection, age >65 years, and diabetes mellitus could be significant predictors for persistent IM or progression to dysplasia. Persistent *H*. *pylori* infection was significantly associated with not only the development of IM but also the progression from IM to dysplasia. Successful *H*. *pylori* eradication is an effective way to induce IM regression and prevent gastric cancer development.

## Supporting information

S1 Table Laboratory results between chronic gastritis without progression and chronic gastritis with progression to IM group (mean ± SD). (DOCX)

S2 Table Risk factors associated with IM status. (DOCX)

S3 Table Laboratory results between IM regression and IM non-regression group (mean ± SD). (DOCX)
