# Supplementary material for: Predictors for regression and progression of intestinal metaplasia (IM): A large population-based study from low prevalence area of gastric cancer (IM-predictor trial)
Source: PLoS One. 2021 Aug 11;16(8):e0255601. doi: 10.1371/journal.pone.0255601 (PMC8357097; doi:10.1371/journal.pone.0255601)
Supplement: S1 Table — (DOCX) [file pone.0255601.s001.docx]

**S1 Table.** Laboratory results between chronic gastritis without progression and chronic gastritis with progression to IM group (mean ± SD)

| **Laboratory results** | **Chronic gastritis**  **non-progression** | **Chronic gastritis progressed to IM** | **P-value** |
| --- | --- | --- | --- |
| Hemoglobin (g/dL) | 11.7 ± 2.4 | 12.0 ± 2.0 | 0.484 |
| WBC count (x10^9^/L) | 7.0 ± 4.3 | 6.8 ± 2.7 | 0.733 |
| Platelet count (x10^9^/L) | 235 ± 119 | 228 ± 65 | 0.579 |
| Creatinine (mg/dL) | 1.1 ± 0.9 | 1.0 ± 0.2 | 0.308 |
| Plasma glucose (mg/dL) | 119 ± 34 | 108 ± 26 | 0.091 |
| Hemoglobin A1C (%) | 6.7 ± 1.8 | 6.2 ± 0.9 | 0.140 |
| Cholesterol (mg/dL) | 175 ± 65 | 192 ± 46 | 0.365 |
| Triglyceride (mg/dL) | 122 ± 59 | 92 ± 29 | 0.097 |
| HDL (mg/dL) | 52 ± 17 | 59 ± 16 | 0.181 |
| LDL (mg/dL) | 99 ± 49 | 108 ± 35 | 0.489 |

WBC = White blood cell, HDL = High-density lipoprotein, LDL = Low-density lipoprotein
