# Supplementary material for: Predictors for regression and progression of intestinal metaplasia (IM): A large population-based study from low prevalence area of gastric cancer (IM-predictor trial)
Source: PLoS One. 2021 Aug 11;16(8):e0255601. doi: 10.1371/journal.pone.0255601 (PMC8357097; doi:10.1371/journal.pone.0255601)
Supplement: S2 Table — (DOCX) [file pone.0255601.s002.docx]

**S2 Table.** Risk factors associated with IM status

| **IM status** | **Negative**  **Hp** | **Eradicated Hp** | | **Persistent**  **Hp** | **OR** | **(95% CI)*** | **p value*** |
| --- | --- | --- | --- | --- | --- | --- | --- |
| **IM regression** | 60.4% | 62.2% | | 39.4% | 0.09 | (0.01-0.85) | 0.035 |
| **IM persistence** | 37.5% | 37.8% | | 45.5% | 1.86 | (0.72-4.79) | 0.200 |
| **IM progression** | 2.1% | 0% | | 15.2% | 11.15 | (1.18-105.24) | 0.035 |
| **IM status** | **No DM** | | **DM** | | **OR** | **(95% CI)** | **p value** |
| **IM regression** | 64.1% | | 35.0% | | 0.37 | (0.17-0.81) | 0.012 |
| **IM persistence** | 33.6% | | 57.5% | | 2.71 | (1.24-5.90) | 0.012 |
| **IM progression** | 2.3% | | 7.5% | | 5.06 | (0.86-29.89) | 0.073 |
| **IM status** | **≤65 years** | | **>65 years** | | **OR** | **(95% CI)** | **p value** |
| **IM regression** | 68.2% | | 46.5% | | 0.40 | (0.21-0.76) | 0.005 |
| **IM persistence** | 29.4% | | 48.8% | | 2.49 | (1.31-4.73) | 0.005 |
| **IM progression** | 2.4% | | 4.7% | | 2.85 | (0.48-17.07) | 0.251 |

Hp = *H. pylori* infection, DM = Diabetes mellitus

*Odds ratios and p values between negative Hp and persistent Hp group
