# Supplementary material for: Predictors for regression and progression of intestinal metaplasia (IM): A large population-based study from low prevalence area of gastric cancer (IM-predictor trial)
Source: PLoS One. 2021 Aug 11;16(8):e0255601. doi: 10.1371/journal.pone.0255601 (PMC8357097; doi:10.1371/journal.pone.0255601)
Supplement: S3 Table — (DOCX) [file pone.0255601.s003.docx]

**S3 Table.** Laboratory results between IM regression and IM non-regression group (mean ± SD)

| **Laboratory results** | **IM regression** | **IM non-regression** | **P-value** |
| --- | --- | --- | --- |
| Hemoglobin (g/dL) | 12.1 ± 2.1 | 11.8 ± 2.5 | 0.579 |
| WBC count (x10^9^/L) | 7.2 ± 2.7 | 7.0 ± 2.2 | 0.597 |
| Platelet count (x10^9^/L) | 229 ± 83 | 251 ± 105 | 0.195 |
| Creatinine (mg/dL) | 1.2 ± 1.5 | 1.1 ± 0.8 | 0.680 |
| **Plasma glucose (mg/dL)** | **106 ± 20** | **128 ± 39** | **0.008** |
| **Hemoglobin A1C (%)** | **6.0 ± 0.7** | **6.8 ± 1.3** | **0.012** |
| Cholesterol (mg/dL) | 172 ± 33 | 169 ± 46 | 0.742 |
| Triglyceride (mg/dL) | 118 ± 55 | 127 ± 62 | 0.578 |
| HDL (mg/dL) | 54 ± 16 | 56 ± 19 | 0.713 |
| LDL (mg/dL) | 99 ± 24 | 99 ± 31 | 0.968 |

WBC = White blood cell, HDL = High-density lipoprotein, LDL = Low-density lipoprotein
